# Pyrenoid loss in *Chlamydomonas reinhardtii* causes limitations in CO_2_ supply, but not thylakoid operating efficiency

**DOI:** 10.1093/jxb/erx197

**Published:** 2017-06-20

**Authors:** Oliver D Caspari, Moritz T Meyer, Dimitri Tolleter, Tyler M Wittkopp, Nik J Cunniffe, Tracy Lawson, Arthur R Grossman, Howard Griffiths

**Affiliations:** 1 Department of Plant Sciences, University of Cambridge, Downing Street, Cambridge, UK; 2 Department of Plant Biology, Carnegie Institution for Science, Stanford, CA, USA; 3 Department of Biology, Stanford University, Stanford, CA, USA; 4 School of Biological Sciences, University of Essex, Wivenhoe Park, Colchester, UK

**Keywords:** Carbon-concentrating mechanism, *Chlamydomonas*, *reinhardtii*, chlorophyll fluorescence, chloroplast, electrochromic shift, electron transport rate, green algae, photosynthesis, pyrenoid, Rubisco

## Abstract

The pyrenoid of the unicellular green alga *Chlamydomonas reinhardtii* is a microcompartment situated in the centre of the cup-shaped chloroplast, containing up to 90% of cellular Rubisco. Traversed by a network of dense, knotted thylakoid tubules, the pyrenoid has been proposed to influence thylakoid biogenesis and ultrastructure. Mutants that are unable to assemble a pyrenoid matrix, due to expressing a vascular plant version of the Rubisco small subunit, exhibit severe growth and photosynthetic defects and have an ineffective carbon-concentrating mechanism (CCM). The present study set out to determine the cause of photosynthetic limitation in these pyrenoid-less lines. We tested whether electron transport and light use were compromised as a direct structural consequence of pyrenoid loss or as a metabolic effect downstream of lower CCM activity and resulting CO_2_ limitation. Thylakoid organization was unchanged in the mutants, including the retention of intrapyrenoid-type thylakoid tubules, and photosynthetic limitations associated with the absence of the pyrenoid were rescued by exposing cells to elevated CO_2_ levels. These results demonstrate that Rubisco aggregation in the pyrenoid functions as an essential element for CO_2_ delivery as part of the CCM, and does not play other roles in maintenance of photosynthetic membrane energetics.

## Teaser

The *Chlamydomonas reinhardtii* pyrenoid is a Rubisco-containing microcompartment primarily responsible for facilitating carbon accumulation and does not affect thylakoid membrane photosynthetic energetics.

## Introduction

The pyrenoid of the model green alga *Chlamydomonas reinhardtii* has long been implicated in a number of functions, most notably the establishment of a carbon-concentrating mechanism (CCM; Badger *et al.*, 1980; Wang *et al.*, 2015). The pyrenoid may also be needed to maintain structural aspects of the chloroplast, including thylakoid membrane organization and translation of proteins and their assembly into complexes, which was found to be localized to specialized zones at the pyrenoid periphery (Uniacke and Zerges, 2007, 2009). Disruption of these pyrenoid-associated processes could potentially impact chloroplast energetics and thus photosynthetic efficiency.

CCMs have arisen multiple times, mostly in aquatic photosynthetic organisms, as a means of increasing carbon fixation under conditions in which CO_2_ availability limits turnover of the Calvin–Benson–Bassham cycle (CBBC; Raven *et al.*, 2012; Meyer *et al.*, 2016). Active transport is used to concentrate dissolved inorganic carbon around the primary carboxylase Rubisco, which allows for more rapid ribulose bisphosphate carboxylation and limits photorespiratory activity, leading to an increase in the ratio of carboxylation to oxygenation. For the CCM to function in *Chlamydomonas*, Rubisco must be confined to a chloroplast microcompartment, the pyrenoid. Assembly of Rubisco into a pyrenoid is controlled by the linker protein EPYC1 (Mackinder *et al.*, 2016), previously designated LCI5 (Lavigne *et al.*, 2001), and two Rubisco small subunit (RBCS) surface α-helices (Meyer *et al.*, 2012). In *Chlamydomonas* RBCS substitution strains expressing vascular plant *RBCS*, Rubisco fails to assemble into pyrenoids, rendering these pyrenoid-less (*pyr–*) cells unable to establish a functional CCM (Genkov *et al.*, 2010; Meyer *et al.*, 2012).

The pyrenoid may also play a role in processes other than the CCM, as suggested by the finding that it is not completely eliminated from wild-type (WT) cells when the CCM is repressed (Borkhsenious *et al.*, 1998). While WT cells redistribute a significant fraction of Rubisco throughout the chloroplast stroma in CCM-repressive conditions, such as at elevated CO_2_ or in the dark, at least 50% of cellular Rubisco remains aggregated in the pyrenoid at all times (Borkhsenious *et al.*, 1998; Mitchell *et al.*, 2014). Additionally, several proteins unrelated to the CCM have been localized to pyrenoids, including nitrite reductase (Süss *et al.*, 1995) and nucleic acid processing enzymes (Shukla *et al.*, 2012; Zhan *et al.*, 2015). Eliminating pyrenoid formation may also alter thylakoid membrane structure since distinct thylakoid domains intersect and coalesce within the pyrenoid (Ohad *et al.*, 1967; Goodenough and Levine, 1970; Engel *et al.*, 2015). Finally, the dispersion of Rubisco throughout the stroma might be expected to favour entropically mediated thylakoid stacking (Chow, 1999; Kim *et al.*, 2005). Indeed, initial observations (Genkov *et al.*, 2010; Meyer *et al.*, 2012) suggested that thylakoid membrane hyperstacking occurred in *pyr–* cells, perhaps reflecting altered photosynthetic energetics in the absence of a functional pyrenoid (Goodenough and Levine, 1969; Chow *et al.*, 2005; Anderson *et al.*, 2008).

The aim of the present study was to understand whether the reduction in growth and photosynthesis, when pyrenoid formation was compromised, was caused directly by the loss of inorganic carbon accumulation capacity, or was also associated with altered thylakoid organization and therefore reduced photosynthetic energetic efficiency. Three *pyr–* lines, generated by complementing a *Chlamydomonas* mutant lacking both native *RBCS* genes with genes encoding vascular plant RBCS (*Spinacia*, *Helianthus*, and *Arabidopsis*; Genkov *et al.*, 2010), were used in the present study. The degree of thylakoid stacking was examined via electron microscopy to determine whether the absence of a pyrenoid altered thylakoid ultrastructure. Furthermore, photosynthetic activity was analysed using a combination of advanced spectroscopic techniques and chlorophyll (Chl) fluorescence measurements. Ultrastructure and whole-cell physiology were studied under contrasting light and CO_2_ regimes.

A sole CCM defect limiting the photosynthetic activity of *pyr*– mutants can be distinguished from additional energetic limitations acting in concert, based on the physiological response of cells to various light and CO_2_ regimes. Production of the high energy metabolites NADPH and ATP via the photosynthetic electron transport chain needs to be balanced with their consumption via the CBBC (Allen, 2003; Eberhard *et al.*, 2008; Foyer *et al.*, 2012; Lane, 2014). If CBBC consumption is reduced because the CCM is absent or impaired, it is reasonable to assume that upstream activity of the electron transport chain would be restricted and the cells would experience a higher photon load than in the presence of a CCM. Excess energy would probably be dissipated via non-photochemical quenching (NPQ), which can be monitored by analysing Chl fluorescence (Krause and Weis, 1991; Lazár, 1999; Maxwell and Johnson, 2000; Papageorgiou and Govindjee, 2004). A photosynthetic defect caused by the loss of the CCM should be minimal at low light, when the supply of photon energy becomes more rate limiting than the supply of CO_2_ (von Caemmerer, 2013; McGrath and Long, 2014), and any difference between mutants and the WT should be abolished at elevated CO_2_ (Fukuzawa *et al.*, 2001; Xiang *et al.*, 2001; Jungnick *et al.*, 2014). In contrast, increased thylakoid stacking or defects in photosynthetic protein complex assembly in the *pyr–* mutants may act to decrease light absorption and thus electron transport chain activity (Falkowski and Raven, 2007), rendering *pyr–* more susceptible to light limitation. In this case, differences from the WT should be exacerbated at low light, and persist in the presence of elevated CO_2_.

The work presented in this study shows that the pyrenoid can be functionally defined as a CCM component (helping supply a high concentration of CO_2_ to aggregated Rubisco). The *pyr*– mutants were not impaired in structural or energetic features based on rescue of all *pyr–* phenotypes once a sufficient supply of CO_2_ was provided to the cells externally. These findings go hand in hand with novel proteomic work on the same mutants (Mitchell *et al.*, 2017) showing that the effect of pyrenoid absence is limited to the CCM and downstream metabolism, with the large majority of proteins expressed at the same levels in the pyrenoid mutants and WT.

## Materials and methods

### Strains and culture conditions

*Pyr–* lines were previously generated by expressing the vascular plant *RBCS* gene, from either *Spinacia oleracea*, *Helianthus annus*, or *Arabidopsis thaliana*, in the ∆∆-*RBCS1,2* host *Chlamydomonas* strain CC-4415 (devoid of the two native RBCS proteins as well as of the state transition regulatory kinase STT7); the control WT cells express the *Chlamydomonas* native *RBCS1* from the same construct in the same ∆∆-*RBCS1,2* host (Genkov *et al.*, 2010). An additional pyrenoid-forming line was provided by the HelixAB strain, which makes a chimeric RBCS containing the *Chlamydomonas* amino acid sequence for the surface α-helices within *Spinacia* RBCS (Meyer *et al.*, 2012). The resulting holoenzymes assemble into a pyrenoid, but display impaired Rubisco kinetic properties, including a 10-fold reduction in the maximum carboxylation rate.

Cells were grown under continuous illumination at 25 °C in Tris-minimal medium (Spreitzer and Mets, 1981). Generally, cultures were maintained under 5% CO_2_ and subjected to experimental CO_2_ conditions overnight prior to any measurements. Standard conditions are defined here as growth at standard light (SL, 50 µmol photons m^−2^ s^−1^) and acclimation to air levels of CO_2_ (0.04%) for ≥12 h. Experimental treatments included growth at low light (LL, 10 µmol photons m^−2^ s^−1^) or higher light (HL, 100 µmol photons m^−2^ s^−1^), and/or with 5% CO_2_ supplementation. Cells were harvested for experiments from mid-log phase cultures, which were at ~2 × 10^6^ cells ml^−1^ or 3–4 µg Chl ml^−1^; Chl levels were quantified according to Wellburn (1994).

To establish growth rates (Fig. 5A), three biological replicates for the WT (taken from two independent *Chlamydomonas RBCS1* insertion lines) and *pyr–* (one expression line each using the *Spinacia*, *Helianthus*, and *ArabidopsisRBCS* constructs) were maintained in 50 ml of liquid medium in experimental conditions (SL/5% CO_2_, SL/air, LL/air) for 6 d in continuous culture. Every 1.5 d, Chl concentrations were measured and cultures were either diluted with fresh medium or supplemented with additional cells from a different culture, in order to attain cultures with mid-log phase growth densities by the next measurement.

### Electron microscopy

WT and *pyr–* (*Spinacia RBCS* strain) samples were fixed for electron microscopy as previously described (Genkov *et al.*, 2010), except that Tris-minimal medium was used instead of PIPES for the first fixation step. Material was imaged using a Field Emission scanning electron microscope (FEI Verios 460L) or a Tecnai G2 80–200 kV transmission electron microscope. Using the latter, 20 cells per experimental condition, each with diameters >4.5 µm, were sampled randomly for quantification of thylakoid stacking. Images were indexed, pooled, and presented in random order for blind analysis. For each image, widths of five representative appressed regions were quantified as the width perpendicular to the orientation of lamellae, using the image processing system Fiji (Schindelin *et al.*, 2012). Statistical analyses of these—and other—results were performed using R version 3.2.4 (The R Foundation, Vienna, Austria).

### Chl fluorescence from algal colonies on solid agar medium

To generate algal colonies on solid agar medium, cultures grown in the dark in liquid Tris-acetate phosphate medium (Spreitzer and Mets, 1981) to a density of ~1.5 µg Chl ml^−1^ were concentrated to 100 µg Chl ml^−1^ in liquid Tris-minimal medium. Replicates of 20 µl were spotted in random order into individual wells of cell culture plates (24 wells) containing solidified Tris-minimal medium +1.5% (w/v) Bacto-Agar. After 4 d of growth under the experimental conditions (LL or SL, air or 5% CO_2_), the cells were dark-adapted for at least 2 h during transport between Cambridge and Colchester. Experimental gas treatments were sustained over the course of the Chl fluorescence measurements, which were made using a Chl fluorescence imaging system (CFimager, Technologica Ltd, Colchester, UK). In order to capture a range of photosynthetic characteristics rapidly, fluorescence parameters were recorded during an initial 20 min fluorescence induction at 106 µmol photons m^−2^ s^−1^, followed by a 5 min dark period to gauge NPQ relaxation, and finally a light intensity response curve (22, 46, 106, 170, 251, 356, 509, and 679 µmol photons m^−2^ s^−1^), with each intensity step lasting 2 min. For presenting the information, PSII operating efficiency (ϕ _II_) values were transformed to electron transport rates (ETRs) as ETR=ϕ _II_×*I*×*a*_II_, where *I* is the light intensity to which the cells were exposed, and *a*_II_ is assumed to be 0.84 × 0.5=0.42, accounting for light absorption (0.84) and distribution between the photosystems (0.5), respectively (Baker, 2008).

A suitable form of the mathematical model proposed by Suggett *et al.* (2003; Equation 1) was fitted to the light response data using Bayesian methods. In its original form, the Suggett *et al.* (2003) model (Equation 1) describes the change in ϕ_II_ in response to changes in *I* in terms of the model parameters ϕ_m_ and *I*_k_, where ϕ_m_ is the maximum PSII operating efficiency and *I*_k_ is the light intensity at which photosynthesis becomes light saturated.

$$\phi_{\text{II}}=\phi_{\text{m}}\frac{I_{k}}{I}\;\left(1-{\text{e}}^{{}^{-\;\frac{I}{I_{k}}}}\right)$$ (1)

Given that ϕ _m_=σ _II_/*a*_II_ (Suggett *et al.*, 2011) and that *I*_k_ is defined as *I*_k_=ETR_max_/σ _II_ (Blackman, 1905), it follows that ϕ _m_×*I*_k_=ETR_max_/*a*_II_ . The relationship can thus be re-expressed in terms of the maximum electron transport rate (ETR_max_; Equation 2), a form that can be more easily interpreted in the context of ETR data (Fig. 2).

$$\phi_{\text{II}}=\frac{{\text{ETR}}_{\text{max}}}{0.42\times I}\;\left(1-{\text{e}}^{{}^{-\frac{I}{I_{k}}}}\right)$$ (2)

The version of the model shown in Equation 2 was fitted to ϕ_II_ data by assuming the experimental data points were normally distributed around a deterministic skeleton from the model, allowing for different values of the parameters ETR_max_ and *I*_k_ for each experimental treatment. The likelihood of the data was then calculated by assuming that all data points were independent, treating the variance of the normal error as an additional nuisance parameter to be estimated (separately for each treatment).

Bayesian methods were used to estimate log-transformed values of model parameters, assuming uninformative improper priors in all cases. The joint posterior of the parameters was estimated using the freely available function MCMCpack (Martin *et al.*, 2011) in R, taking 1 000 000 samples after discarding the first 20 000 as burn in. Proper convergence was assessed by visual inspection of trace plots and by checking that results were unaffected by chain initial conditions. Credible intervals for the mean response of WT and *pyr–* to light in terms of ETR were calculated—at each value of the independent variable—by considering the distribution formed by taking 10 000 samples from the joint posterior distribution and using them in Equation 2, and then transforming the predicted ϕ_II_ values to ETR as described above.

To test whether model parameters differed between pairs of experimental treatments, posterior probability distributions were estimated for differences in parameters between pairs of experiments (i.e. ΔETR_max_ or Δ*I*_k_) using 100 000 pairs of samples from the posterior distributions. Since this procedure leads to a full probability distribution, it can be used to estimate 95% confidence intervals (CIs) for any difference in parameter values, as well as the probability that the difference in parameters is less than zero (Kruschke, 2011). In interpreting the results, it should be noted that the latter probability would be 0.5 if there were absolutely no difference between the two treatments, meaning that—to the extent it is possible to compare the Bayesian and frequentist inferential frameworks—any value <0.025 is equivalent to a ‘significant’ difference.

### Spectroscopy

For measurements using the Joliot-type spectrophotometer (JTS-10, Bio-Logic Science Instruments SAS, Claix, France), cells grown in liquid medium were suspended in 20 mM HEPES (pH 7.2) containing 10% (w/v) Ficoll at a density of 10–12 µg Chl ml^−1^. Where applicable, cultures were split after dark adaptation and half of the culture was supplemented with sodium bicarbonate to a final concentration of 10 mM. Chl fluorescence parameters were estimated using standard procedures (Maxwell and Johnson, 2000) and functional PSI/PSII ratios were estimated from the electrochromic shift (ECS) absorbance (520 nm) using single turnover flashes (Joliot and Delosme, 1974; Bailleul *et al.*, 2010). For a subset of experiments, xenon bulb flashes were used, which were later estimated to have resulted in 1.74 PSI turnovers on the basis of laser flash recordings. Additionally, ECS traces were used to estimate total electron flow rate (TEF; Joliot and Joliot, 2002; Bailleul *et al.*, 2010; Lucker and Kramer, 2013). Fluorescence saturation was measured in samples treated with the PSII inhibitor DCMU (20 µM) to obtain a measure of the functional absorption cross-section of PSII based on the time taken to reach two-thirds of maximal fluorescence (*t*_2/3_). In the presence of the PSII inhibitors hydroxylamine (1 mM) and DCMU, P700 oxidation–reduction kinetics were recorded for quantification of total PSI (Hiyama and Ke, 1972; Alric, 2010; Johnson and Alric, 2012), which was used to calculate Chl per PSII. This analysis takes into account the ECS-based PSI/PSII ratio and the total Chl content of the cells, and assumes that each PSI contains 240 Chls (Drop *et al.*, 2014). Alternatively, Chl per PSII was estimated from the Chl *a*/*b* ratio according to Drop *et al.* (2014), assuming a PSI/II ratio of 1.

The same Bayesian approach was used to fit ETR, TEF, and NPQ light response curves as described above for Chl fluorescence data from algal colonies, similarly using the model shown in Equation 2 on ϕ_II_ data on which the ETR curves are based. This relationship was used to derive a modified version for TEF, based on the definition of ETR and assuming ETR≈TEF, as shown in Equation 3.

$$\text{TEF}={\text{TEF}}_{\text{max}}\;\left(1-{\text{e}}^{-\frac{I}{I_{k}}}\right)$$ (3)

$$\text{NPQ}={\text{NPQ}}_{\text{max}}\frac{I^{n}}{I_{50}^{\text{n}}+I}$$ (4)

Equation 4 shows the model used for NPQ data (Serôdio and Lavaud, 2011), where NPQ_max_ is the maximum level NPQ that can be attained, *I*_50_ is the light intensity at which half-maximal NPQ is reached, and *n* is the Hill coefficient that controls the sigmoidicity of the curve.

## Results

### Thylakoid membrane organization is not affected by absence of the pyrenoid

**Fig. 1.**
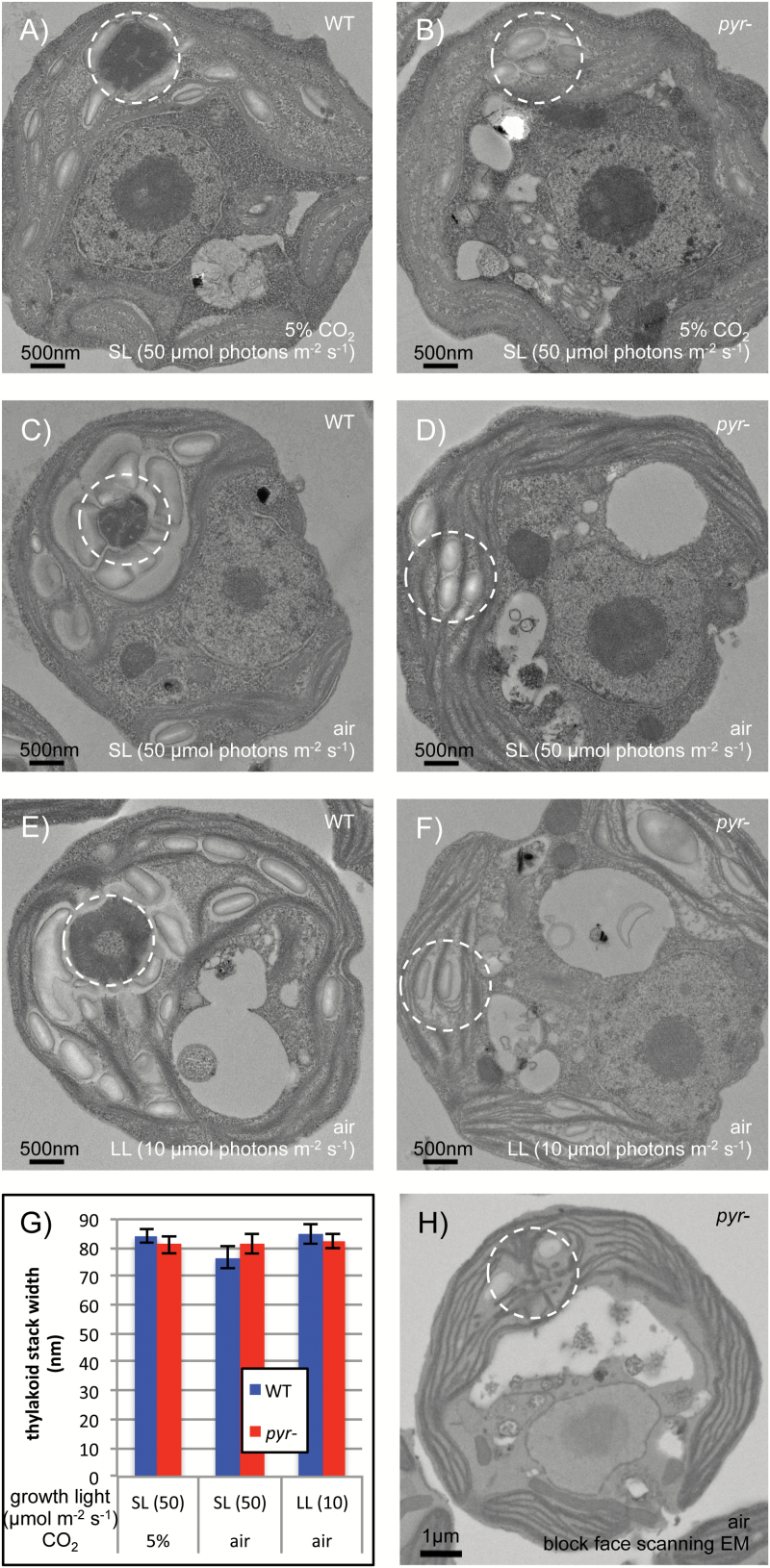
Pyrenoid absence has no effect on thylakoid arrangement. TEM shows WT and *pyr–* (*Spinacia RBCS*) cells grown in contrasting physiological conditions in (A–F) with the canonical pyrenoid location circled. Low light (LL) was 10 µmol photons m^−2^ s^−1^ and standard light (SL) was 50 µmol photons m^−2^ s^−1^. A quantification of thylakoid stacking is provided in terms of the width of appressed regions (G) as mean ±SE across 20 cells per condition (five measurements per cell). Block face SEM (H) reveals that knotted thylakoid tubules are retained in *pyr–*.

TEM was used to investigate any effect of the pyrenoid on thylakoid ultrastructure (Fig. 1). In WT cells, the pyrenoid is an electron-dense body positioned at the base of the cup-shaped chloroplast and surrounded by a starch sheath (Fig. 1A, C, E). *Pyr*– cells exhibit starch granules in the region where the pyrenoid usually assembles (Fig. 1B, D, F). Thylakoids appear as single lamellae or appressed in stacks that often run parallel to the chloroplast envelope and occupy most of the stroma. The extent of stacking, quantified by measuring the width of several appressed regions per cell across 20 randomly chosen cells, was found to be very similar beween the WT and *pyr–* (Fig. 1G; *P*=0.912).

WT pyrenoids are traversed by a complex network of modified thylakoid tubules that fuse at the centre of the microcompartment (Ohad *et al.*, 1967; Goodenough and Levine, 1970; Engel *et al.*, 2015). Block face SEM revealed that this network is retained in *pyr–* mutants (Fig. 1H, see Supplementary Fig. S1 at *JXB* online for further images). The tubules appear to meet at the same position as in WT cells, close to where the centre of the pyrenoid would have been. Other than the absence of the pyrenoid matrix, chloroplast ultrastructure thus appears unaltered in *pyr–* cells.

### *Differences in ETR between the WT and* pyr– *are cancelled when CO_2_ is not limiting*

**Fig. 2.**
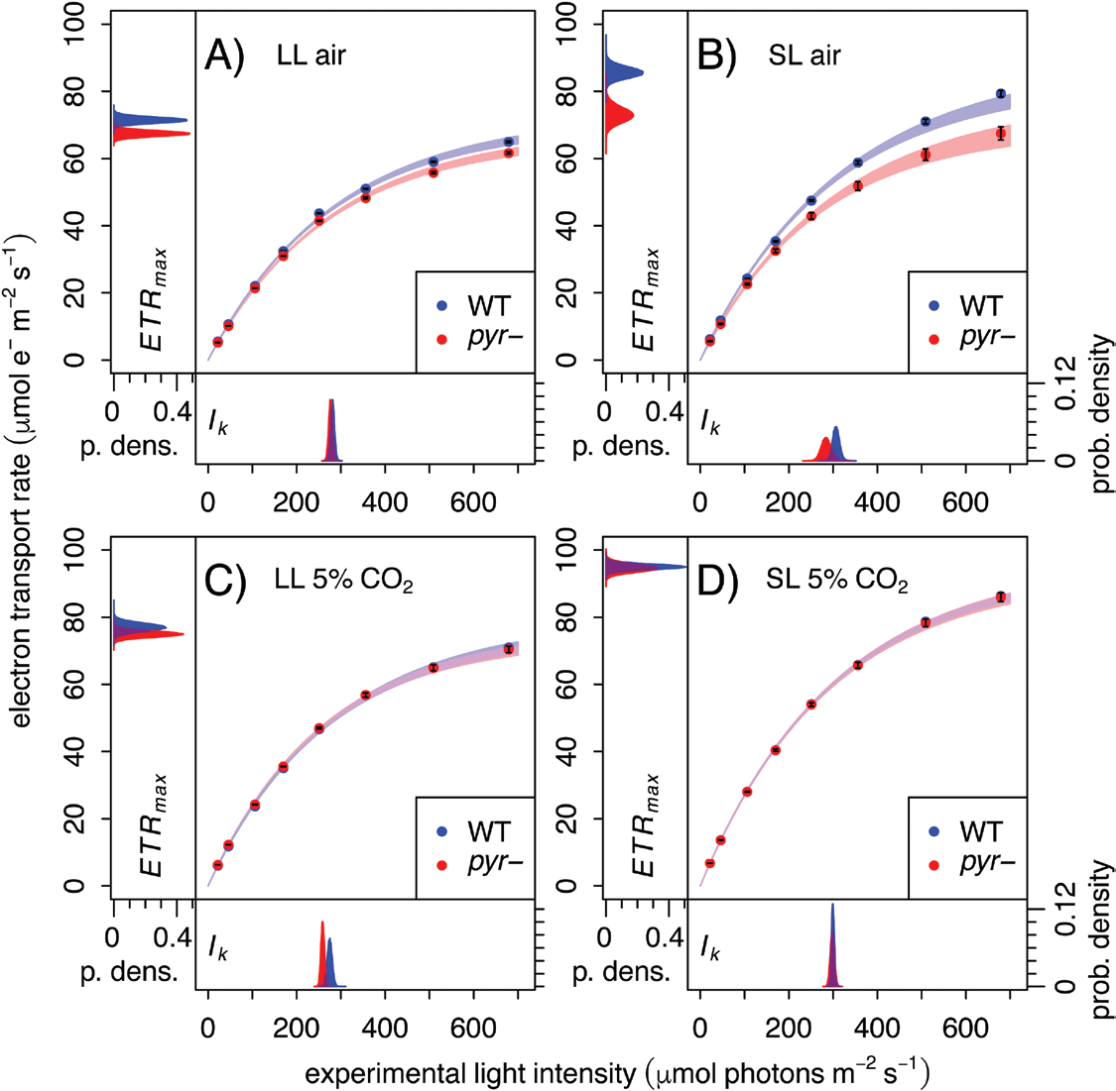
Under CCM-inducing conditions, *pyr–* shows a reduction in CO_2_-limited photosynthesis (ETR_max_). Light response curves were collected using a Technologica CFimager from algal colonies grown on agar plates in LL (low light, 10 µmol photons m^−2^ s^−1^) or SL (standard light, 50 µmol photons m^−2^ s^−1^) in air or 5% CO_2_, as indicated. The ETR is shown as the mean of six biological replicates ±SE, overlaid with 95% confidence intervals derived from fitting a light response model (Equation 2). Probability density plots of marginal posteriors for the fit parameters ETR_max_ and *I*_k_ are shown in the figure margins.

For an initial investigation into the nature of photosynthetic energetics in the absence of a functional pyrenoid, fluorescence parameters were estimated from WT and *pyr*– colonies grown on agar in contrasting light and CO_2_ regimes and subjected to actinic light intensities of 20–700 µmol photons m^−2^ s^−1^ (Fig. 2). Use of a camera-based system ensured that the majority of the signal originates from the topmost layer of cells that have plentiful access to light and air. At ambient CO_2_, the *pyr*– mutant showed lower ETR through PSII than the WT (Fig. 2A, B). This impact was most pronounced when cells had been grown at SL (50 µmol photons m^−2^ s^−1^; Fig. 2B) rather than LL (10 µmol photons m^−2^ s^−1^; Fig. 2A), consistent with limited induction of the CCM in the latter condition as light is more limiting than CO_2_. In contrast, ETR was similar for WT and *pyr–*cells at 5% CO_2_ (Fig. 2C, D).

These effects were quantified via statistical analysis, with the primary data fitted using a Bayesian approach and the mathematical relationship proposed by Suggett *et al.* (2003). Model parameters ETR_max_ and *I*_k_ report on the CO_2_-limited maximum rate of electron transport and the light saturation point, respectively. Fitted values and uncertainties are shown as marginal posterior densities in the plot margins (Fig. 2). The difference between the air-acclimated WT and the *pyr–* mutant (Fig. 2A, B) could be accounted for mostly in terms of ETR_max_, a direct proxy for CO_2_ limitation [*P*(ΔETR_max_≤0)_LL_=4.5 × 10^–4^; *P*(ΔETR_max_≤0)_SL_=2 × 10^–5^]. The difference in ETR_max_ was reduced in LL (Fig. 2A) [95% CI, CI(ΔETR_max_)_LL_, 1.6–6.3 µmol m^−2^ s^−1^; 95% CI(ΔETR_max_)_SL_, 6.8–18.2 µmol m^−2^ s^−1^] and eliminated at 5% CO_2_ (Fig. 2C, D) [*P*(ΔETR_max_≤0)_LL_=0.093; *P*(ΔETR_max_≤0)_SL_=0.391].

### *The* pyr– *phenotype is akin to a reduced Rubisco carboxylation rate*

**Fig. 3.**
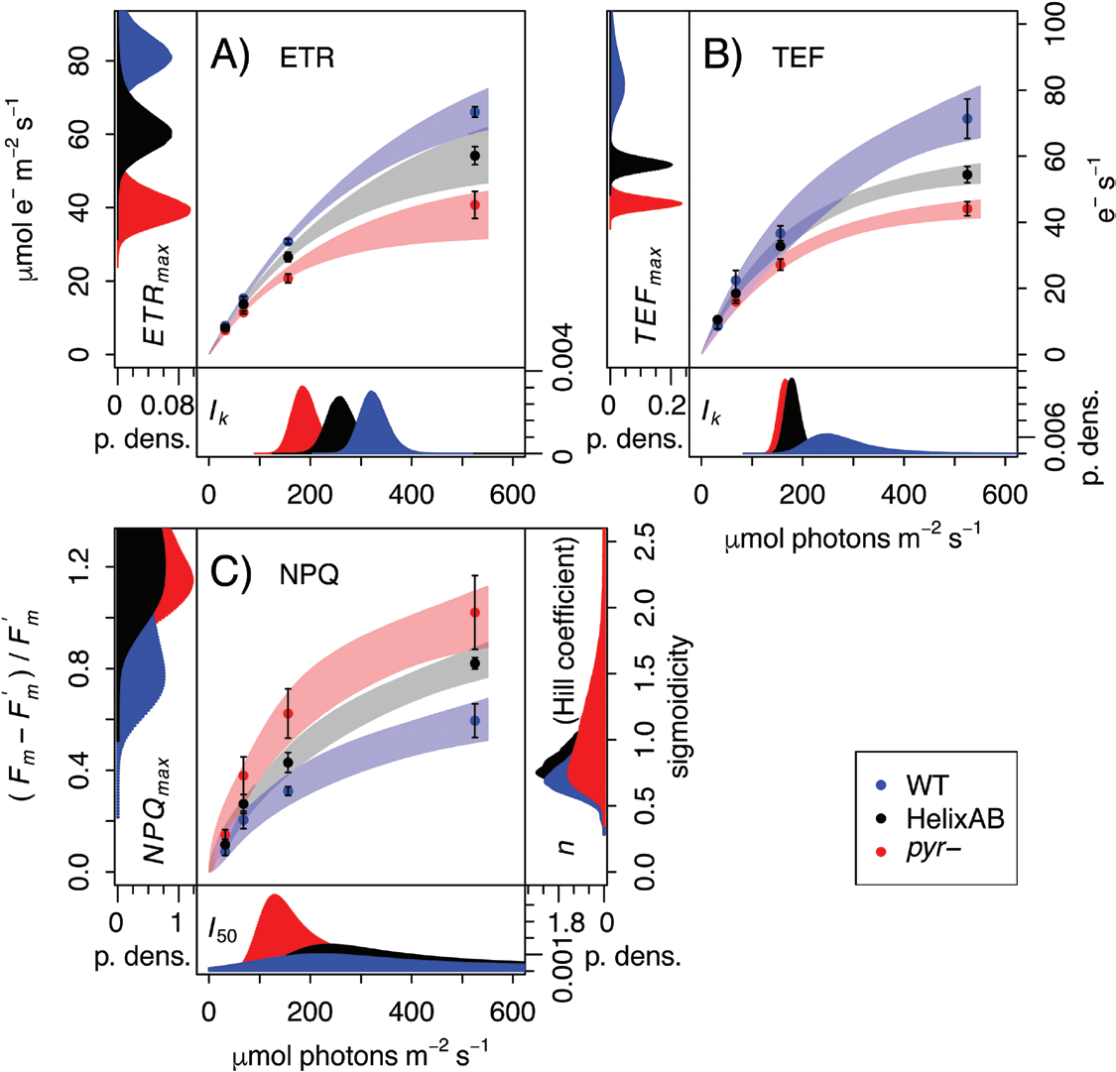
High-resolution ECS and Chl fluorescence measurements reveal feedback limitation by Rubisco catalysis. Panels show JTS-10 data of (A) ETR through PSII estimated on the basis of Chl fluorescence data, (B) TEF estimated on the basis of ECS data, and (C) NPQ. Data are the mean ±SE based on ≥3 biological replicates each, overlaid with 95% confidence intervals derived using a Bayesian approach to capture the underlying physiological dynamics in terms of model parameters. ETR_max_, TEF_max_, and NPQ_max_ describe the maximum attainable levels of each photosynthetic indicator. *I*_k_ is the light saturation point, while *I*_50_ is the light half-saturation point. Finally, *n* is the Hill coefficient which controls the sigmoidicity of the NPQ curve, and can be indicative of allosteric regulation when *n*>1. Probability density plots of the marginal posteriors for fit parameters are shown in the figure margins. Cells were grown in liquid culture in the absence of aeration at 50 µmol photons m^−2^ s^−1^.

The difference in photosynthetic activity between air-acclimated *pyr*– and the WT under SL was confirmed using spectroscopic approaches in liquid cultures (Joliot and Joliot, 2002; Joliot *et al.*, 2004). This allowed the ETR through PSII (Fig. 3A) to be quantified alongside whole-chain TEF rates (Fig. 3B) based on the ECS method (Joliot and Delosme, 1974; Bailleul *et al.*, 2010). As observed for agar-grown colonies (Fig. 2B), *pyr–* strains exhibited significantly lower rates of photosynthetic electron transport [*P*(ΔETR_max_≤0)=0; *P*(ΔTEF_max_≤0)=0] as well as increased NPQ (Fig. 3C).

For comparison with *pyr*–, a second mutant defective for CO_2_ fixation, designated HelixAB, was characterized. This strain does form a pyrenoid but suffers from impaired Rubisco kinetic properties owing to the chimeric nature of the RBCS in that strain (Meyer *et al.*, 2012). Like *pyr–*, HelixAB consistently showed significant impairment of TEF and ETR (Fig. 3A, B) relative to the WT [*P*(ΔETR_max_≤0)=1.8 × 10^–2^; *P*(ΔTEF_max_≤0)=6 × 10^–5^] as well as increased NPQ (Fig. 3C). That the phenotype exhibited by *pyr–* is similar to that of HelixAB, which is limited by the enzymatic activity of Rubisco, suggests that the *pyr–* defect results from a similarly reduced CBBC turnover as a consequence of being unable to supply saturating CO_2_ to Rubisco.

**Fig. 4.**
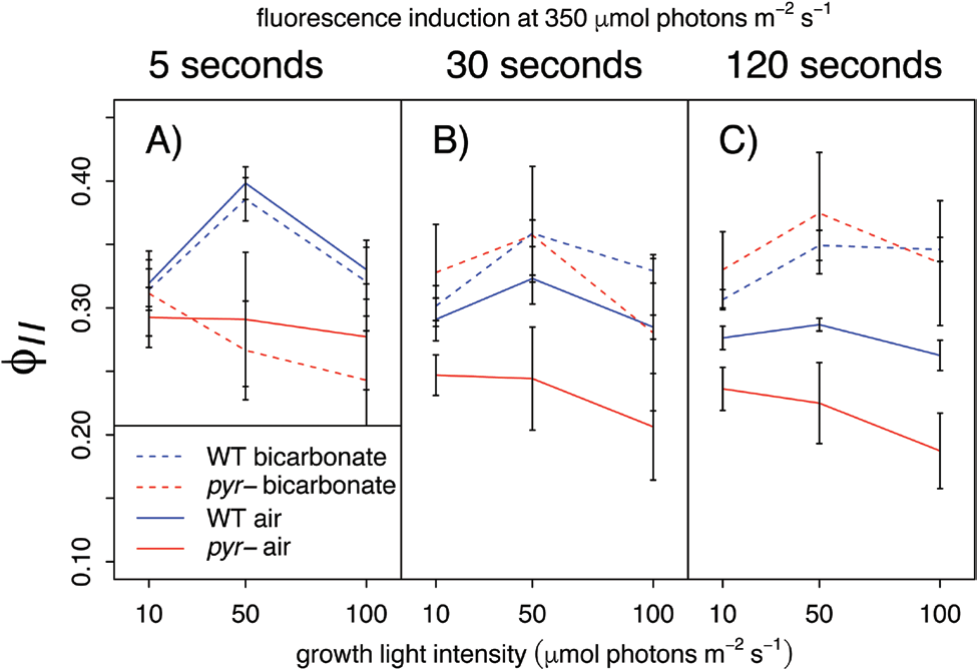
Addition of bicarbonate restores photosynthetic performance in air-acclimated *pyr–* cells. PSII operating efficiency (ϕ _II_) was measured in the JTS-10 after 5, 30, and 120 s at ~350 µmol photons m^−2^ s^−1^ (to ensure saturation; see *I*_k_; Figs 2, 3) as detailed above the data panels. Cells were grown in air at a range of light intensities (LL, SL, and HL) as shown on the *x*-axis. Cultures supplemented with 10 mM sodium bicarbonate directly before measurements are shown by dashed lines. Data are the mean of three biological replicates ±SE.

Elevated CO_2_ restores *pyr–* photosynthetic performance via CBBC feedback: To determine the short-term response to elevated CO_2_, saturating levels were generated by the addition of sodium bicarbonate (10 mM final, equivalent to aeration with ≥1% CO_2_) during spectroscopic measurements of a fluorescence induction time-course at saturating light (350 µmol photons m^−2^ s^−1^; cf. *I*_k_, Figs 2, 3). In the absence of bicarbonate, air-acclimated *pyr*– cells showed lower ϕ _II_ than the WT (Fig. 4A–C, solid lines, *P*=1.46 × 10^–5^), in line with the previous results (Figs 2, 3). In the presence of bicarbonate (dashed lines), *pyr*– samples exhibited values of ϕ _II_ comparable with those of WT cells (*P*=0.674) following ≥30 s of illumination (Fig. 4B, C, dashed lines). An initial >5 s lag (Fig. 4A) reappears after each short dark incubation in repeated measurements on the same cells (data not shown), ruling out the idea that the effect is due to a slow entrance of bicarbonate into the cells. Rather, the lag is consistent with activation of the CBBC, which has been well characterized in fluorescence induction experiments (Krause and Weis, 1991; Lazár, 1999; Maxwell and Johnson, 2000; Papageorgiou and Govindjee, 2004). These results thus suggest that the limitation observed in the mutant cells is imposed by slow CBBC turnover under low CO_2_, which is overcome when saturating levels of CO_2_ are supplied externally.

### *Changes in photosystem and accessory pigment composition occur in both the WT and* pyr– *during acclimation*

**Fig. 5.**
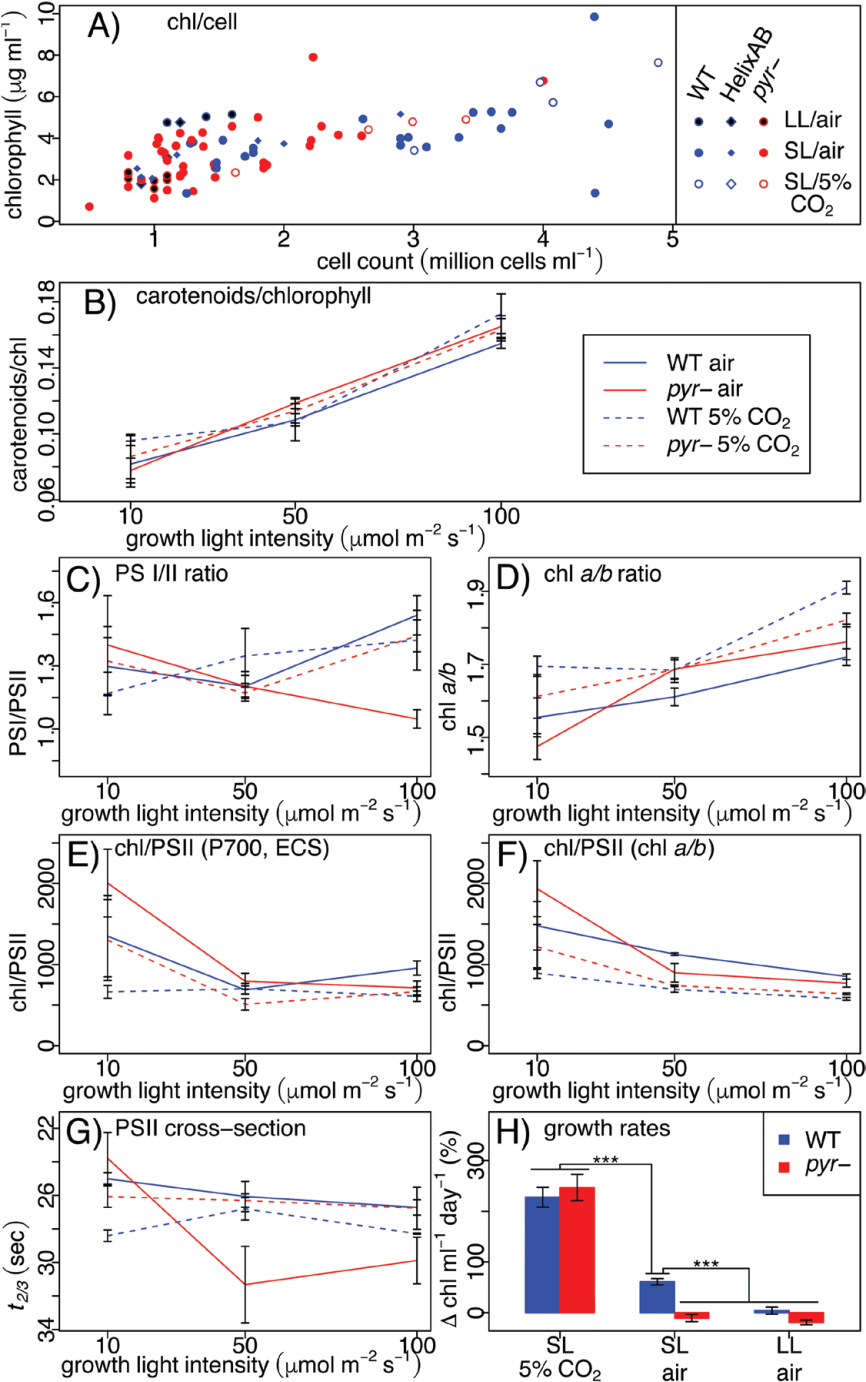
Accumulation of pigments undergoes physiological acclimation independent of pyrenoid phenotype. Chl expression on a per cell basis (A) is shown as a scatterplot of individual measurements. Starting with the accumulation of carotenoids relative to Chl (B), data are plotted against growth light intensity and shown as the mean of three biological replicates ±SE. The functional PSI/PSII ratio (C) and the Chl *a*/*b* ratio (D), respectively, enable quantification of Chl allocation to PSII from spectroscopic data (E) and Chl extraction data (F). Fluorescence saturation kinetics in the presence of DCMU (*t*_2/3_) provide a proxy for functional PSII antenna size (G). Growth rates (H) are shown as percentage change in Chl ml^−1^ over 24 h as mean ±SE based on four measurements over the course of 6 d of three biological replicates per condition grown in quasi-continuous culture; three asterisks signify statistical significance at the level of *P*<0.01 estimated through ANOVA.

Levels of Chl, carotenoids, and the photosystems were quantified for the same cultures used for the fluorescence induction time-course measurements (Fig. 4). The WT and *pyr–* exhibited an equivalent relationship between Chl content and culture density (Fig. 5A, *P*=0.232). The ratio of carotenoids to Chl (Fig. 5B), quantified according to Wellburn (1994), increased significantly (*P*=2.85 × 10^–9^) with growth light intensity, but was also similar between the WT and *pyr–* (*P*=0.38). PSI/PSII ratios (Fig. 5C) were generally similar in WT and mutant cells under the different growth conditions used (*P*>0.3), except possibly for air-acclimated cells at HL (100 µmol photons m^−2^ s^−1^; *P*=2.1 × 10^–14^). Levels of Chl *a* relative to Chl *b* (Fig. 5D) were highest in HL (*P*=1.5 × 10^–5^) and, again, similar in the WT and *pyr–* (*P*=0.503). Consistently, the allocation of Chl to PSII decreased at higher light intensities (*P*=2.1 × 10^–14^) when either measured spectroscopically (Fig. 5E) or inferred from Chl extraction data (Fig. 5F), calculated according to Drop *et al.* (2014), with no significant difference between the WT and *pyr*- (*P*=0.616).

In contrast, functional association of Chl and PSII (Fig. 5G), measured as PSII absorption cross-section (*t*_2/3_, an inverse proxy; see the Materials and methods), was reduced in air-acclimated *pyr–* as compared with the WT (*P*=2.33 × 10^–3^) at both SL and HL. No such difference was observed for cells grown at LL (*P*=0.245) or 5% CO_2_ (*P*>0.999). A similar pattern was observed for growth rates, quantified as percentage change in Chl ml^−1^ d^−1^ (Fig. 5H), which differed significantly between air-acclimated WT and *pyr–* at SL (*P*=0.009), but not when the light intensity was reduced to LL (*P*=0.86) or the CO_2_ concentration increased to 5% (*P*=0.94). Thus *pyr–* cells experience the same changes as the WT in their accumulation of photosynthetic pigments during acclimation to the various growth light regimes, despite marked differences in their growth rates when exposed to low levels of CO_2_.

## Discussion

### Absence of the pyrenoid matrix has no effect on thylakoid ultrastructure

In contrast to our initial hypothesis, a quantitative assessment revealed no difference in thylakoid membrane stacking between *pyr–* and the WT under any of the growth conditions (Fig. 1G). This finding is at odds with the idea that the Rubisco aggregation state could play a role in determining thylakoid stacking via a free energy trade-off (Chow, 1999; Kim *et al.*, 2005). Rather, control of thylakoid stacking appears to lie with lateral heterogeneity of the photosystems: PSII-associated light-harvesting complex II (LHCII) subunits foster cation-modulated attraction between lamellae in flowering plants (Chow *et al.*, 2005; Anderson *et al.*, 2008), whereas PSI impedes stacking through steric hindrance, which explains the hyperstacking phenotype in PSI-deficient *Chlamydomonas* mutants (Goodenough and Levine, 1969).

Additionally, the complex network of modified thylakoids normally associated with pyrenoids (Ohad *et al.*, 1967; Engel *et al.*, 2015) was retained in *pyr–* mutants (Fig. 1H; Supplementary Fig. S1). This finding is in accord with previous observations that knotted tubules persist in mutants that retain only a very reduced pyrenoid (Ma *et al.*, 2011; Mackinder *et al.*, 2016) as well as in a mutant devoid of Rubisco due to reduced levels of chloroplast ribosomes (Goodenough and Levine, 1970). Since *pyr–* cells behave as WT except for a defect in the CCM, any CCM-unrelated proteins that localize to the pyrenoid in the WT, such as nitrite reductase or nucleic acid processing enzymes (Süss *et al.*, 1995; Shukla *et al.*, 2012; Zhan *et al.*, 2015), must be functioning normally in the absence of a pyrenoid matrix, and may even be targeted to the same location in *pyr–* cells. Similarly, there was no significant difference in accumulation of pigments during acclimation to the different growth light regimes between the WT and *pyr–* (Fig. 5). Synthesis and assembly of photosynthetic protein complexes, which occurs in pyrenoid-associated translation zones in the WT (Uniacke and Zerges, 2007, 2009), thus probably also functions normally in *pyr–* cells [unless a growth arrest (Fig. 5H) is masking a defect confined to low CO_2_]. Consistently, proteomic work by Mitchell *et al.* (2017), published in this issue, found the abundances of the large majority of proteins unaffected by pyrenoid phenotype.

Despite the inability of *pyr–* to aggregate Rubisco into a pyrenoid matrix, pyrenoid-associated non-matrix structures thus seem to form and function as in WT cells. Rubisco aggregation is known to be controlled by two RBCS surface helices (Meyer *et al.*, 2012) and the linker protein EPYC1 (Mackinder *et al.*, 2016), formerly designated LCI5 (Lavigne *et al.*, 2001). It thus seems likely that the vascular plant RBCSs expressed in *pyr–* lines are simply unable to interact with EPYC1, in the context of an otherwise normal chloroplast. Through an interaction between EPYC1/LCI5 and thylakoid membranes (Turkina *et al.*, 2006), thylakoid tubules may act as an anchor point around which the pyrenoid matrix assembles (Goodenough and Levine, 1970). However, starch accumulates in *pyr–* at the canonical pyrenoid location without obviously interacting with the knotted thylakoid tubules (Fig. 1B, D, F). Thus thylakoid-independent mechanisms must exist that target chloroplast components to the pyrenoid location.

### Pyr– *cells are CO_2_ limited*

When light is plentiful, photosynthesis is generally limited by CO_2_ supply, whereas at low light the supply of photons becomes rate limiting (Eberhard *et al.*, 2008; Foyer *et al.*, 2012; von Caemmerer, 2013; McGrath and Long, 2014). That air-acclimated WT and *pyr–* phenotypes were similar at LL, but not SL (growth, Fig. 5H; ETR, Fig. 2A, B, ϕ_II_, Fig. 4) was thus a first indication that *pyr–* cells are simply limited by CO_2_, in line with the established CCM defect (Genkov *et al.*, 2010; Meyer *et al.*, 2012). HelixAB, a strain that forms a pyrenoid but expresses a kinetically impaired chimeric Rubisco (Meyer *et al.*, 2012), shows a similar difference from the WT (Fig. 3), further supporting the notion that the *pyr–* defect is a consequence of the slower turnover rate of the CBBC.

The capacity to absorb light, but not process all of the incoming energy via the CBBC, means that air-acclimated *pyr–* cells should have a greater need to dissipate excess energy. Indeed, photoprotective NPQ was found to be higher (Fig. 3C) and the functional PSII absorption cross-section lower (Fig. 5G) in air-acclimated *pyr–* than in the WT in SL. Such a reduction in cross-section could temporarily reduce light absorption and be under the control of cellular regulation. Increased photoinhibition in *pyr–* may also contribute to a lower PSII absorption cross-section and increased NPQ. Since the *STT7* kinase was deleted alongside *RBCS1* and *2* in the genetic host used to generate the strains studied here (Khrebtukova and Spreitzer, 1996), state transitions do not play a role.

The addition of high CO_2_ facilitated a complete recovery of *pyr–* photosynthetic characteristics to those of WT cells, not just after long term-growth at 5% CO_2_ (ETR, Fig. 2C, D; growth, Fig. 5H; PSII cross-section, Fig. 5G) but within seconds of illumination following the addition of saturating levels of bicarbonate (Fig. 4B, C). The photosynthetic electron transport chain of air-acclimated *pyr–* cells is thus just as competent to process incoming light as that of the WT. Therefore, *pyr–* strains are bona fide CCM mutants (Fukuzawa *et al.*, 2001; Xiang *et al.*, 2001; Jungnick *et al.*, 2014), with photosynthetic impairments the sole consequence of limitations in the supply of CO_2_. That Rubisco must be aggregated for the CCM to function (Genkov *et al.*, 2010; Meyer *et al.*, 2012) implies a role for the pyrenoid matrix in limiting back-diffusion of CO_2_ (Meyer *et al.*, 2016). Through the current work, this microcompartment is now functionally fully defined as a component of the CCM.

## Supplementary data

Supplementary data are available at *JXB* online.

Fig. S1. Further block face SEM images of intrapyrenoid-like thylakoid tubules in *pyr–* cells.

## Author contributions

Electron microscopy was performed by MTM and analysed by ODC; the Chl fluorescence imaging experiment was designed by ODC, TL, and HG, and performed by ODC; model fitting and comparison for the Chl fluorescence imaging data was designed by NJC and refined by ODC; ECS and Chl fluorescence light response curves recorded in the JTS-10 were designed by ODC, DT, ARG, and TMW, and performed and analysed by ODC; the bicarbonate addition experiment recorded in the JTS-10 was designed by ODC and ARG, and performed and analysed by ODC; growth and pigment data were collected by ODC. ODC wrote the manuscript; all authors read and commented on the manuscript and approved the final version.

## Supplementary Material

supplementary_figure_S1

## Abbreviations:

CBBC: Calvin–Benson–Bassham cycle
CCM: carbon-concentrating mechanism
Chl: chlorophyll
CI: confidence interval
ECS: electrochromic shift
ETR: electron transport rate
HL: high light (100 µmol photons m^−2^ s^−1^)
ETR_max_: maximum electron transport rate
*I*_k_: parameter describing the minimum light intensity that saturates photosynthesis
LL: low light (10 µmol photons m^−2^ s^−1^)
NPQ: non-photochemical quenching
ϕ_II_: PSII operating efficiency
pyr–: pyrenoid-less
RBCS: Rubisco small subunit
SL: standard light (50 µmol photons m^−2^ s^−1^)
TEF: total electron flow
WT: wild type.
