# Supplementary material for: Pyrenoid loss in Chlamydomonas reinhardtii causes limitations in CO2 supply, but not thylakoid operating efficiency
Source: J Exp Bot. 2017 Jun 20;68(14):3903–13. doi: 10.1093/jxb/erx197 (PMC5853600; doi:10.1093/jxb/erx197)
Supplement: supplementary_figure_S1 [file erx197_suppl_supplementary_figure_s1.pdf]

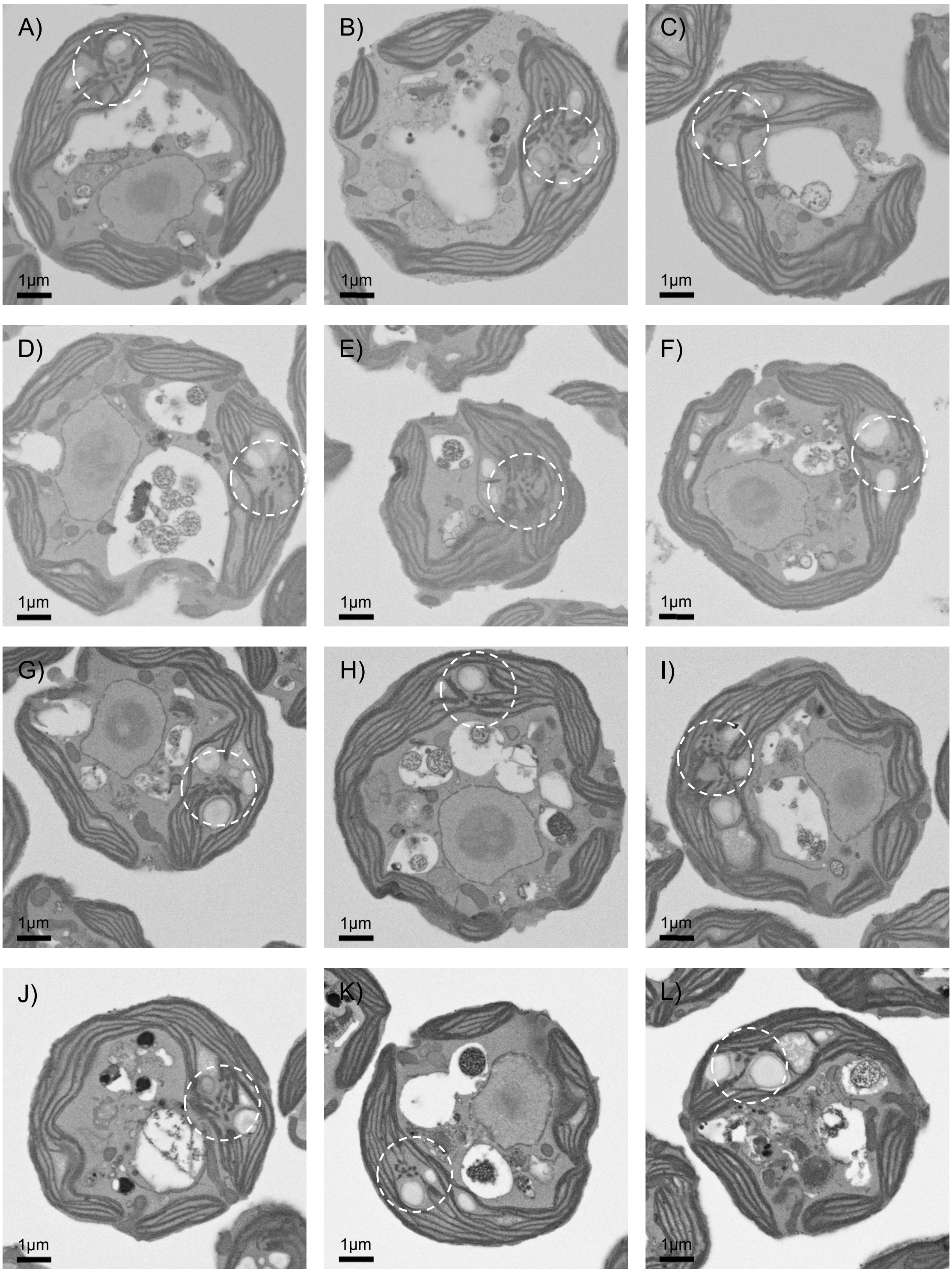

S1: Knotted thylakoid tubules persist in the absence of a pyrenoid

Block face scanning electron microscopy images show *pyr*- cells (*Spinacia RBCS*) grown at air. A circle highlights the location where the pyrenoid would normally be expected in a WT cell. For comparison with the main text, (A) is the same as Fig. 1H.
